# Detection of spotted fever group rickettsiae and *Coxiella burnetii* in long-tailed ground squirrels (*Spermophilus undulatus*) and their ectoparasites

**DOI:** 10.3389/fvets.2025.1553152

**Published:** 2025-03-06

**Authors:** Xiaoshuang Han, Ziheng Liu, Zhixian Jiang, Shanshan Zhao, Sándor Hornok, Meihua Yang, Gang Liu, Yuanzhi Wang

**Affiliations:** ^1^Key Laboratory for Prevention and Control of Emerging Infectious Diseases and Public Health Security, the XPCC, School of Medicine, Shihezi University, Shihezi, Xinjiang, China; ^2^NHC Key Laboratory of Prevention and Treatment of Central Asia High Incidence Diseases, First Affiliated Hospital, School of Medicine, Shihezi University, Shihezi, Xinjiang, China; ^3^Department of Forest, Agriculture College, Shihezi University, Shihezi, Xinjiang, China; ^4^Department of Parasitology and Zoology, University of Veterinary Medicine, Budapest, Hungary; ^5^HUN-REN-UVMB Climate Change: New Blood-Sucking Parasites and Vector-Borne Pathogens Research Group, Budapest, Hungary

**Keywords:** *Spermophilus undulatus*, flea, louse, spotted fever group rickettsiae (SFGR), *Coxiella burnetii*

## Abstract

Long-tailed ground squirrels (LTGRs, *Spermophilus undulatus*) are known as reservoirs of multiple arthropod-borne pathogens, such as *Yersinia pestis* and *Bartonella rochalimae*. However, data on the prevalence of spotted fever group rickettsiae (SFGR) and *Coxiella burnetii* in LTGRs and its ectoparasites are limited. In two alpine regions of Xinjiang Uygur Autonomous Region (XUAR, northwestern China), a total of 346 samples were collected from 142 LTGRs, including 142 livers and 204 pooled ectoparasites (*Citellophilus tesquorum dzetysuensis*: 120 pools of 484 fleas; *Frontopsylla elatoides elatoides*: 19 pools of 71 fleas; *Neopsylla mana*: 1 pool of 4 fleas; and *Linognathoides urocitelli*: 64 pools of 865 lice). From these samples, the DNA was extracted, followed by PCR amplification of different genetic markers. Particularly, genes encoding the outer membrane protein A and B (*ompA*, *ompB*), citrate synthase (*gltA*), and surface cell antigen 1 (*sca1*) were used to identify the SFGR. Additionly, the capsular outer membrane protein (*Com1*) gene and insertion sequence (*IS1111*) genes were used to detect *Coxiella*. *Rickettsia sibirica* subsp. *sibirica*, *Rickettsia felis*, and *C. burnetii* were detected in LTGRs, as well as in flea and louse pools. *Rickettsia raoultii* was found in LTGRs and flea pools. Furthermore, *Rickettsia slovaca* was also identified in the flea pools. This study provides molecular evidence for the occurrence of SFGR and *C. burnetii* in LTGRs and their ectoparasites. These findings suggest that *R. sibirica*, *R. slovaca*, *R. raoultii*, *R. felis* and *C. burnetii* are transmitted between LTGRs (as potential reservoirs) and their fleas and lice (as potential vectors).

## 1 Introduction

Species of the genus *Rickettsia* belong to four distinct phylogenetic clades: spotted fever group, typhus group, ancestral group, and transitional group (1). The spotted fever group rickettsiae (SFGR) include over 30 distinct species that may cause severe infections in humans, domestic animals and wildlife (2). The majority of SFGR are tick-borne, while *R. felis* is typically transmitted by fleas. Notably, some SFGR species can be transmitted by lice and mosquitoes, albeit rarely (3–5).

*Coxiella burnetii*, recognized as the causative agent of Q fever, stands out as the most notorious member of its genus (6). As a globally distributed pathogen, it can infect a diverse range of mammals, from rodents to bats (7, 8). The main vectors of *C. burnetii* are hard ticks (7), but it is also potentially transmitted by fleas and lice (9, 10).

Long-tailed ground squirrels (LTGRs, *Spermophilus undulatus*) are medium-sized ground-dwelling rodents, inhabiting distinct alpine habitats in Central Asia, including Kazakhstan, Mongolia, the southern region of the Russian Federation and northwestern China (11). Previously, LTGRs and arthropods infesting them were shown to act as reservoirs or potential vectors for some pathogens, such as *Yersinia pestis*, *Borrelia burgdoferi* sense lato, *Anaplasma phagocytophilum*, *Trypanosoma otospermophili*, *Blechomonas luni*, tick-borne encephalitis virus and *Hepacivirus* C (12–16). However, the evidence of SFGR and *C. burnetii* in LTGRs and their ectoparasites, especially in fleas and lice, remains still unknown. The aim of this study was to screen these pathogens in liver samples of LTGRs and their associated arthropods.

## 2 Materials and methods

### 2.1 Sample collection and identification

In total, 142 LTGRs were captured in July, 2024 in Wenquan County and Jinghe County (1200–2,500 m above sea level, both adjacent to Kazakhstan), Bortala Mongolian Autonomous Prefecture, northwestern China (Supplementary Figure 1). To achieve this goal, Sherman traps (H.B. Sherman Traps, Tallahassee, FL, United States) were deployed at the entry points of occupied burrows. The survey encompassed a total of 150 traps per site, which were inspected once an hour (17).

All captured rodents were identified by experienced zoologists based on morphological characteristics, such as body length, fur color, tail length and other features (17). Subsequently, the rodent were euthanized and killed via cervical dislocation by certified personnel at the enhanced biosafety level 2 laboratory, Shihezi University (18). Each sampled rodent was then put in individual ziplock bags stored at −80°C. The liver was removed from individual LTGR and placed into each labeled tubes. The species was confirmed from four liver samples of representative rodents by sequencing the cytochrome b (*cytb*) gene (19). All procedures involving wild rodents adhered to the ethical guidelines of Animal Ethics Committee of Shihezi University (Approval No. A2022-029-01).

Arthropod ectoparasites (559 fleas, 865 lice and 1,136 ticks) were collected from individual rodents through gentle brushing of their fur, and then preserved in 70% ethanol. All sampled ticks were used for virus research by another team. For morphological identification, the fleas and lice were treated with 10% NaOH for 1–3 days and put onto slides for microscopical examination (11, 20). Meanwhile, the cytochrome c oxidase subunit II (*COII*) gene for fleas, and the 18S ribosomal RNA (*18S rRNA*) gene for lice were amplified and sequenced, in order to confirm their taxonomy (21–23). Subsequently, the ectoparasite samples were grouped into pools according to individual host from where they were collected, flea or louse species, number and sampling sites. Flea pools contained 2 to 5 individuals, while lice pools contained 8 to 15 individuals. Finally, fleas were allocated into a total of 140 pools and lice into 64 pools which were used to screen pathogens as described below.

### 2.2 Detection, sequencing and phylogenetic analysis

Each arthropod pool and liver sample were extracted with TIANamp Genomic DNA Kit (TIANGEN, Beijing, China) according to the manufacturer’s instructions. Four genetic markers, including outer membrane proteins A and B (*ompA* and *ompB*), citrate synthase (*gltA*) and surface cell antigen 1 (*sca1*), were used to detect SFGR (17). In addition, the capsular outer membrane protein (*Com1*) gene and the insertion sequence (*IS1111*) gene were targeted to investigate the presence of *C. burnetii* (24, 25). The primer sequences and PCR conditions are shown in Supplementary Tables 1, 2. Negative controls consisted of double-distilled water, which consistently showed no detectable PCR product in all tests. Positive controls were DNA samples of *Rickettsia lusitaniae* from common pipistrelles and *Coxiella*-like symbiont from ticks, both preserved in our laboratory (26, 27). The PCR products were purified using the TIANgel Midi Purification Kit (TIANGEN, Beijing, China), and sequenced with Sanger and 454-pyrosequenced PCR amplicons (28, 29). The above sequencing was conducted three times to check the reproducibility. Obtained sequences were compared to reference sequences found in GenBank using BLAST.^1^ Phylogenetic analysis was conducted using MEGA 7.0 software, employing the neighbor-joining method and 1,000 replicates for bootstrap support.

## 3 Results

### 3.1 Morphological and molecular identification of rodent species and associated fleas and lice

**Figure 1:**
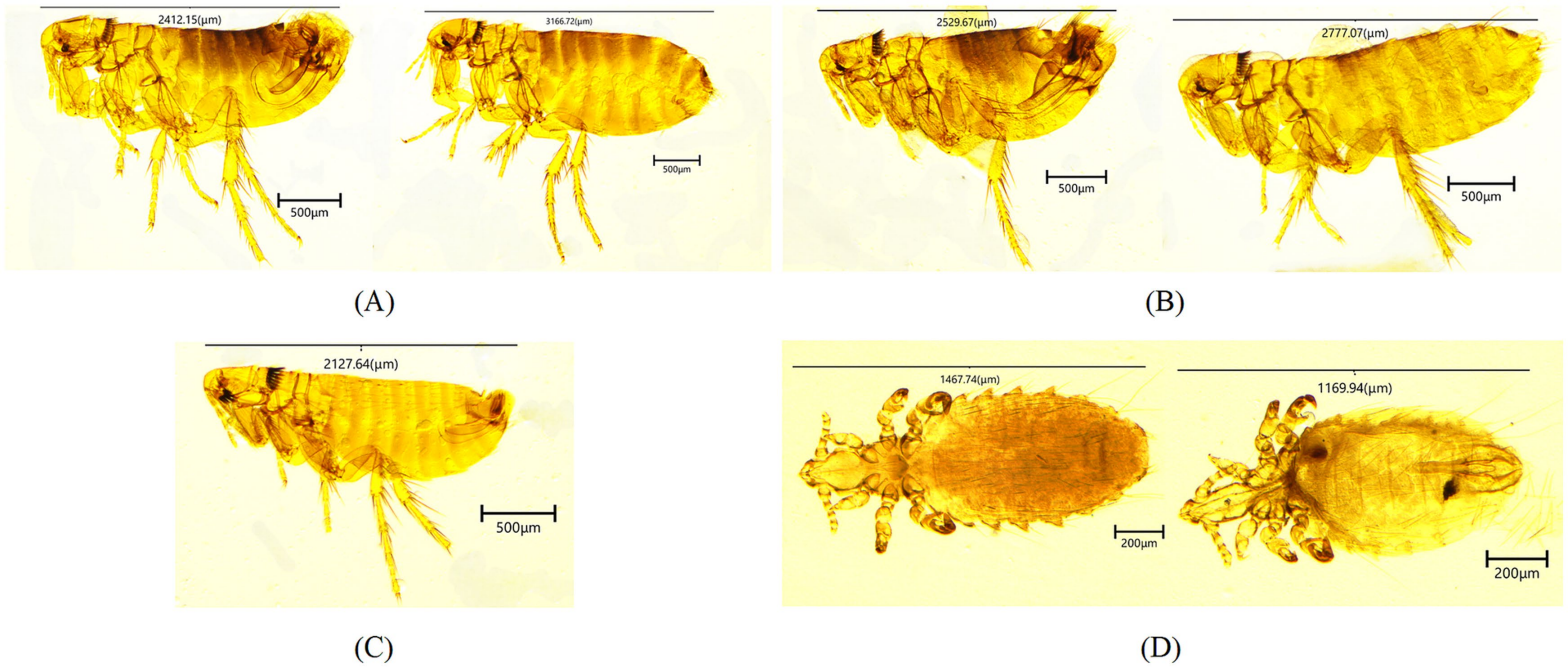
Photomicrographs of morphologically identified fleas and lice [*Citellophilus tesquorum dzetysuensis* **(A)**, *Frontopsylla elatoides elatoides* **(B)**, *Neopsylla mana* **(C)**, *Linognathoides urocitelli* **(D)**].

All sampled rodents were identified as long-tailed ground squirrels (*S. undulatus*) based on their morphology and a 98.86% sequence identity (1,124/1137 bp) to the *cytb* gene of this species found in Russia (OQ695583). A total of 559 fleas were collected. The average flea index was 3.94 (559/142). Subsequently, three species were identified after microscopical examination (Figure 1), including *Citellophilus tesquorum dzetysuensis* (484), *Frontopsylla elatoides elatoides* (71) and *Neopsylla mana* (4). The former flea species was morphologically indientified by genal comb absent, reduced frontal bristles (1 in males, absent in females), pronotal ctenidium with 18–22 vertical spines, labial palps extending to or beyond forecoxa. The middle was by prominent frontal tubercle, 6–7 frontal bristles, 3 ocular bristles above eye, pronotal ctenidium with 22 spines, labial palps reaching forecoxa apex. The latter was by two genal combs (outer comb with short, broad spines; inner comb with narrow, posteriorly inclined spines), pronotal ctenidium with 17–20 spines, labial palps reaching two-thirds of forecoxa. The *COII* gene sequences of these species exhibited 100, 99.86% (713/714 bp), and 100% sequence identities to those of conspecific fleas reported from China, respectively (PP475165, MF000677 and MF000670). Furthermore, 865 lice were also collected. The average louse index was 6.09 (865/142). All sampled lice in this study were identified as *Linognathoides urocitelli* by molecular detection and morphological key features, which included dorsoventrally flattened body, reduced eyes, 5-segmented antennae with sexually dimorphic spines on third segment (males), thoracic sternal plate with posterior medial lobe, and paratergal plates on abdominal segments III-VII bearing spiracular openings. The *18S rRNA* gene sequence of this species exhibited 99.81% (522/523 bp) sequence identity to the same louse species from LTGR in Mongolia (MK478719).

### 3.2 Molecular and phylogenetic analysis of spotted fever group rickettsiae (SFGR)

Eleven LTGRs (7.75%, 11/142), 12 flea pools (8.57%, 12/140) and 15 louse pools (23.44%, 15/64) tested positive for SFGR. Among the 11 SFGR-positive LTGRs, 10 liver samples were also SFGR-positive in their associated flea and louse pools. BLAST and phylogenetic analyses revealed that *R. sibirica* and *R. felis* were present in LTGRs, flea and louse pools. Additionally, *R. raoultii* was identified in LTGRs and flea pools. Moreover, *R. slovaca* was also detected in the flea pools (Table 1).

**Table 1:** Information on specimens used in this study, including geographical location of collection site, total number, pool number, hosts, and prevalence of pathogens.

Location	Species	Number of specimens (pools)	Host species (number)	SFGR	*Coxiella burnetii*	Co-infection
Jinghe County	*Citellophilus tesquorum dzetysuensis*	357 (90)	*Spermophilus undulatus* (90)	5/90 (5.56%) *Rickettsia sibirica*	43/90 (47.78%)	2/90 (2.22%)
				1/90 (1.11%) *Rickettsia slovaca*		–
				3/90 (3.33%) *Rickettsia raoultii*		–
				1/90 (1.11%) *Rickettsia felis*		–
	*Frontopsylla elatoides elatoides*	30 (9)	*Spermophilus undulatus* (9)	1/9 (11.11%) *Rickettsia sibirica*	7/9 (77.78%)	1/9 (11.11%)
	*Neopsylla mana*	4 (1)	*Spermophilus undulatus* (1)	–	1/1 (100%)	–
	*Linognathoides urocitelli*	785 (58)	*Spermophilus undulatus* (58)	12/58 (20.69%) *Rickettsia sibirica*	19/58 (32.76%)	2/58 (3.45%)
				2/58 (3.45%) *Rickettsia felis*		–
	*Spermophilus undulatus*	101 (−)	–	7/101 (6.93%) *Rickettsia sibirica*	9/101 (8.91%)	2/101 (1.98%)
				1/101 (0.99%) *Rickettsia raoultii*		–
				1/101 (0.99%) *Rickettsia felis*		–
Wenquan County	*Citellophilus tesquorum dzetysuensis*	127 (30)	*Spermophilus undulatus* (30)	–	6/30 (20%)	–
	*Frontopsylla elatoides elatoides*	41 (10)	*Spermophilus undulatus* (10)	1/10 (10%) *Rickettsia sibirica*	1/10 (10%)	–
	*Linognathoides urocitelli*	80 (6)	*Spermophilus undulatus* (6)	1/6 (16.67%) *Rickettsia sibirica*	1/6 (16.67%)	–
	*Spermophilus undulatus*	41 (−)	–	2/41 (4.88%) *Rickettsia sibirica*	6/41 (14.63%)	–

**Figure 2:**
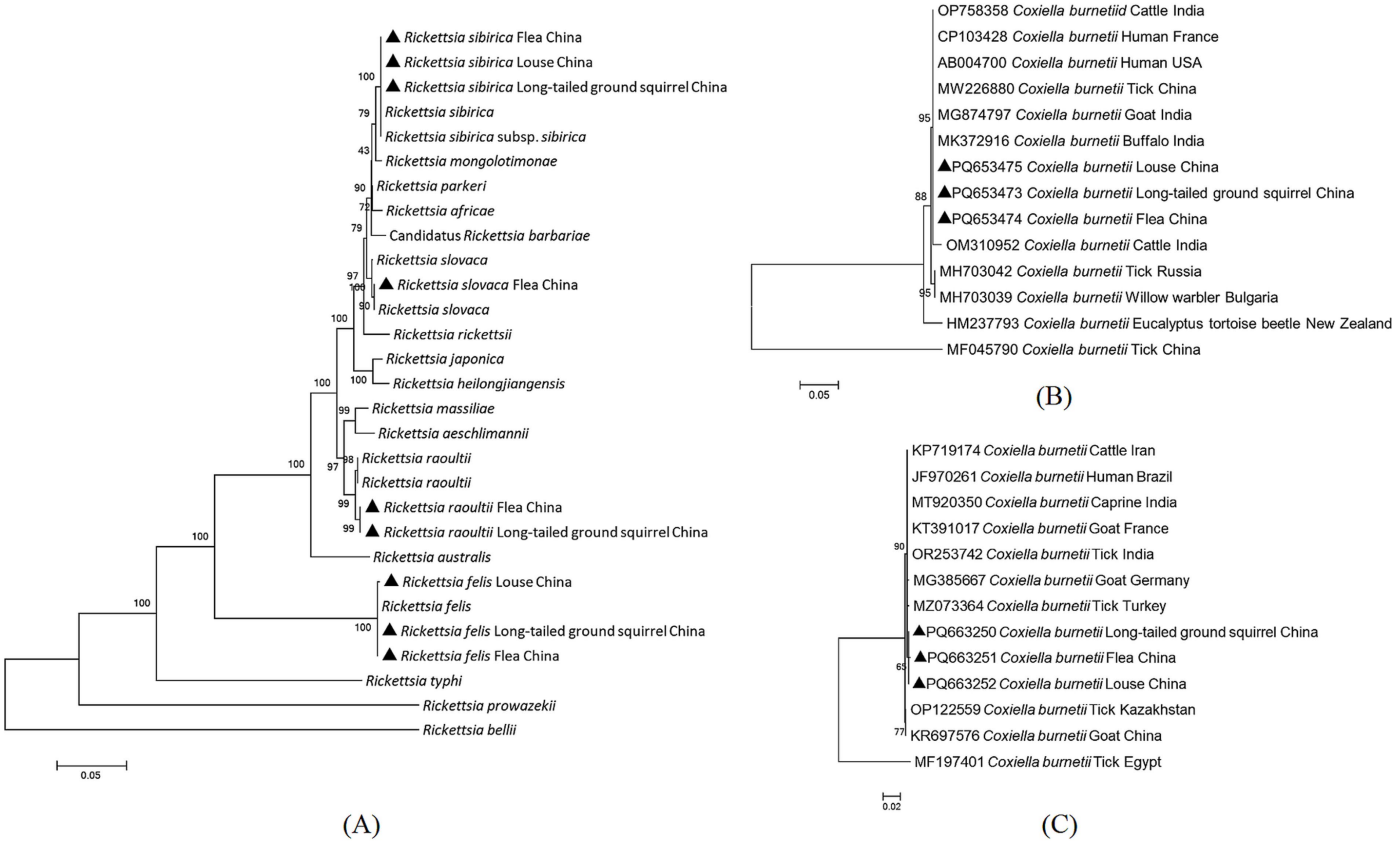
Phylogenic tree of spotted fever group rickettsiae (SFGR) and *Coxiella burnetii* from long-tailed ground squirrels and their ectoparasites [the *ompA*-*ompB*-*gltA*-*sca1* concatenated sequences for SFGR **(A)**, the *Com1* concatenated sequences for *C. burnetii* **(B)**, the *IS1111* concatenated sequences for *C. burnetii* **(C)**]. The new sequences provided in the present study are indicated by a black triangle (containing the accession number).

Regarding sequence comparisons based on the four genetic markers, *Rickettsia sibirica* subsp. *sibirica* showed 100% identity compared with the sequence of conspecific bacteria from a tick-bitten patient in Russia (KT006594); *R. slovaca* showed 100% identity to *R. slovaca* from *Dermacentor marginatus* ticks in Kazakhstan (MW922580); *R. raoultii* showed 99.47–99.50% identity to *R. raoultii* from striped field mouse (*Apodemus agrarius*) in China (MZ297809); and *R. felis* showed 99.76–100% identity compared with the sequence of *R. felis* from cat fleas (*Ctenocephalides felis*) in Indonesia (MT499365; Figure 2). The detailed similarities and divergences of the sequences in this study are shown in Supplementary Table 3.

### 3.3 Molecular and phylogenetic analysis of *Coxiella burnetii*

*C. burnetii* was detected in 15 LTGRs (10.56%, 15/142), 58 flea pools (41.43%, 58/140) and 20 louse pools (31.25%, 20/64). The prevalence of *C. burnetii* in both LTGRs and their ectoparasites was significantly higher than that of SFGR (*X^2^* = 35.09, *df* = 2, *p* < 0.05), suggesting a more prominent circulation of *C. burnetii* in these region. Based on BLASTn analysis, the *IS1111* sequences (PQ663250-PQ663252) in this study were closest related to a *C. burnetii* isolate from *Rhipicephalus sanguineus* ticks in Turkey (MZ073364), showing 99.54–99.69% identities (Figure 2).

### 3.4 Co-infection of *Rickettsia sibirica* and *Coxiella burnetii*

Notably, two LTGRs (1.41%, 2/142) were co-infected with *R. sibirica* and *C. burnetii*. More interestingly, their associated three flea pools (2.14%, 3/140) and two louse pools (3.13%, 2/64) were also co-infected with *R. sibirica* and *C. burnetii*.

## 4 Discussion

In this study, 142 LTGRs and their ectoparasites, 559 fleas and 865 lice were collected in Central Asia. In these samples, four SFGR species were detected, including *R. sibirica*, *R. slovaca*, *R. raoultii*, and *R. felis*, as well as *C. burnetii*. To our best knowledge, (i) *R. sibirica*, *R. felis* and *C. burnetii* were identified here for the first time in LTGRs and their parasites (fleas and lice); (ii) in this study, first molecular evidence is also provided for *R. raoultii* in LTGRs and their ectoparasitic fleas; (iii) additionally, *R. slovaca* is newly recognized in LTGR fleas.

SFGR have worldwide distribution and affect a wide range of wild and domestic vertebrates. Although most reports focus on tick-borne transmission of SFGR, growing evidence suggests that other arthropods-including fleas (notably for *R. felis*), lice, keds, and bugs-may serve as potential vectors (2). *R. felis* is primarily transmitted by fleas, particularly by the cat flea (*C. felis*) (30). Similarly, molecular evidence was provided for *R. slovaca* in *Haematopinus suis* lice from boars in Algeria and later in *Laelaps agilis*, *Laelaps jettmari* and *Eulaelaps stabularis* mites from rodents in Slovakia (31, 32). With regard to *R. raoultii*, its occurrence was detected in fleas from yellow necked mouse (*Apodemus favicollis*) and bank vole (*Myodes glareolus*) in Germany (33). In this study, we reported novel findings of *R. sibirica* and *R. felis* in *C. tesquorum dzetysuensis*, *F. elatoides elatoides* and *L. urocitelli*, as well as *R. slovaca* and *R. raoultii* in *C. tesquorum dzetysuensis* and *F. elatoides elatoides*. In addition, SFGR were previously detected in wild boars, birds, dogs and cats, which act as reservoirs (34–37). Rodents are also notably recognized as reservoirs including yellow necked mouse (*Apodemus flavicollis*), common vole (*Microtus arvalis*) and European water vole (*Arvicola terrestris*) (38). Interestingly, we found that *R. sibirica*, *R. raoultii* and *R. felis* were present not only in the aforementioned arthropods but also in LTGRs. Notably, among 11 SFGR-positive LTGRs, 10 liver samples were also SFGR-positive in their associated flea and louse pools. This high concordance suggests that ectoparasites may play a critical role in either acquiring pathogens from infected hosts or transmitting SFGR within rodent populations. Although we have detected these SFGR in LTGRs and their ectoparasites, further experiments are still needed to confirm the role of these arthropods as vectors.

*C. burnetii*, listed as category B bioterrorism agent in United States, has diverse infection routes including air-borne and venereal transmission (39). Furthermore, it is transmitted by infected arthropods, such as ticks, fleas, lice and mites (10, 40–42). Previously, *C. burnetii* was found in *Haematopinus eurysternus* lice from a cow in Egypt (10). More recently, a report revealed the presence of *C. burnetii* in fleas collected from Norway rat (*Rattus norvegicus*), black rat (*Rattus rattus*) and Cyprus red fox (*Vulpes vulpes indutus*) in Cyprus (43). In this study, we extend these findings with the novel detection of *C. burnetii* in *C. tesquorum dzetysuensis*, *F. elatoides elatoides*, *N. mana* and *L. urocitelli*. Additionally, *C. burnetii* was previously identified in deer, sheep, cattle, goats, birds and rabbits, which serve as reservoirs (7). Rodents also have an important role as reservoirs, as exemplified by the flying squirrel (*Pteromys volans*), red squirrel (*Sciurus vulgaris*), and red-backed vole (*Myodes gapperi*) (44). In our research, LTGRs have been found to harbor *C. burnetii* as well. Crucially, all *C. burnetii*-positive LTGRs in this study concurrently carried infected fleas or lice, suggesting a potential bi-directional transmission between hosts and ectoparasites.

Rodents serve as hosts for several species of blood-sucking arthropods, and infected ectoparasites may transmit pathogens to these mammals through their bites. Consequently, naïve ectoparasites themselves can become infected after biting these rodent reservoirs, leading to new opportunities for pathogens to spread by horizontal transmission (Supplementary Figure 2). Examples of such pathogens include *R. slovaca*, *R. raoultii*, *R. felis* and *R. monacensis* (33, 45). Therefore, the concurrent detection of pathogens in hosts, fleas, and lice is of significant importance, underscoring the complexity of pathogen transmission and the potential roles of these vectors in pathogen spillover and host shift.

## 5 Conclusion

In this study, we present the first molecular evidence of SFGR and *C. burnetii* in LTGRs and their ectoparasites. Our findings suggested that *R. sibirica*, *R. slovaca*, *R. raoultii*, *R. felis* and *C. burnetii* are transmitted between LTGRs (as potential reservoirs) and fleas and lice (as potential vectors) infesting them.

## Data Availability

The datasets presented in this study can be found in online repositories. The names of the repository/repositories and accession number(s) can be found at: https://www.ncbi.nlm.nih.gov/, Rodent species cytb: PQ653469; flea species COII: PQ653470-PQ653472; louse species 18S rRNA: PQ640277; SFGR ompA: PQ677906-PQ677914; SFGR ompB: PQ677915-PQ677923; SFGR gltA: PQ677924-PQ677932; SFGR sca1: PQ677933-PQ677941; Coxiella burnetii Com1: PQ653473-PQ653475; Coxiella burnetii IS1111: PQ663250-PQ663252.

## Footnotes

^1^ http://www.ncbi.nlm.nih.gov/BLAST/

## Supplementary material

SUPPLEMENTARY FIGURE 1 The locations for capturing rodents and associated ectoparasitic arthropods in the current study.

SUPPLEMENTARY FIGURE 2 Circulation of pathogens between ectoparasites and rodents (LTGRs): **(A)** Ectoparasites (e.g., fleas and lice) acquire pathogens by biting infected rodents (LTGRs); **(B)** Pathogen-carrying ectoparasites bite uninfected rodents (LTGRs); **(C)** Uninfected rodents (LTGRs) become infected by bites from pathogen-carrying ectoparasites.

SUPPLEMENTARY TABLE 1 Target genes and sequences of primers used in this study for identifying Long-tailed ground squirrels (LTGRs), fleas, lice, spotted fever group rickettsiae (SFGR) and *Coxiella burnetii*.

SUPPLEMENTARY TABLE 2 Cycling conditions of PCR assays.

SUPPLEMENTARY TABLE 3 The BLAST analysis of Long-tailed ground squirrels (LTGRs), fleas, lice, spotted fever group rickettsiae (SFGR) and *Coxiella burnetii*.
