# Supplementary figures and images for: Detection of spotted fever group rickettsiae and Coxiella burnetii in long-tailed ground squirrels (Spermophilus undulatus) and their ectoparasites
Source: Front Vet Sci. 2025 Mar 6;12:1553152. doi: 10.3389/fvets.2025.1553152 (PMC11923762; doi:10.3389/fvets.2025.1553152)

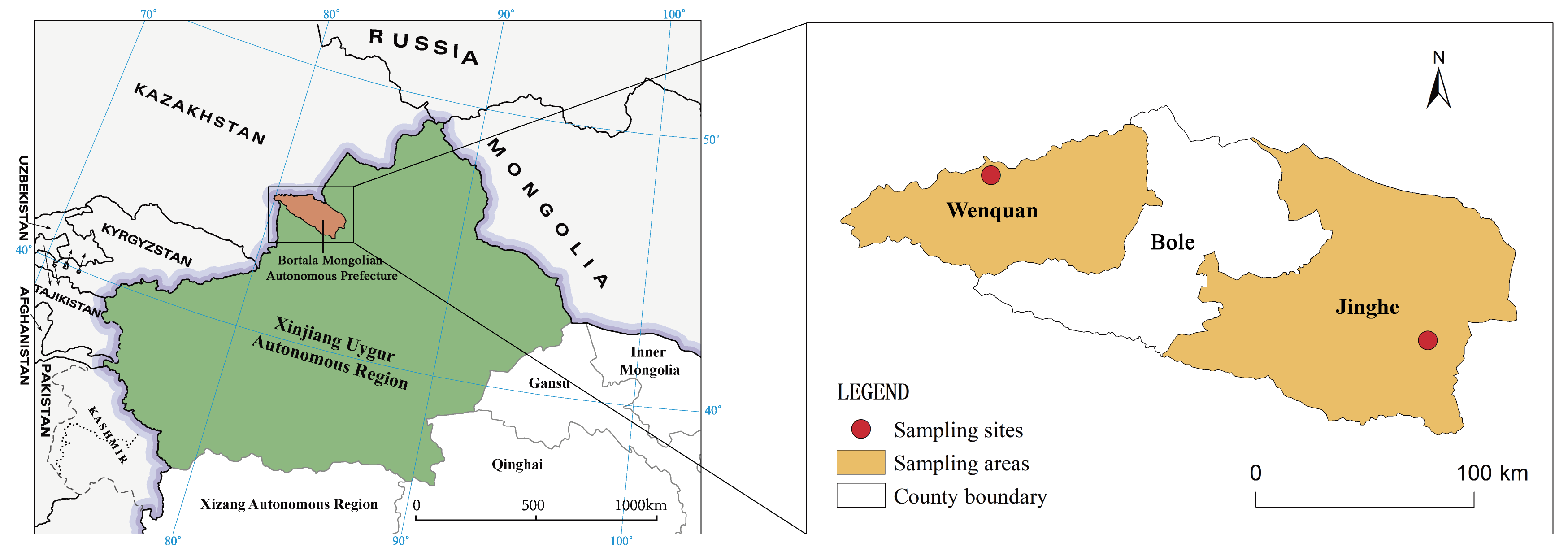

Supplement: SUPPLEMENTARY FIGURE 1 — The locations for capturing rodents and associated ectoparasitic arthropods in the current study. [file Image_1.tiff]

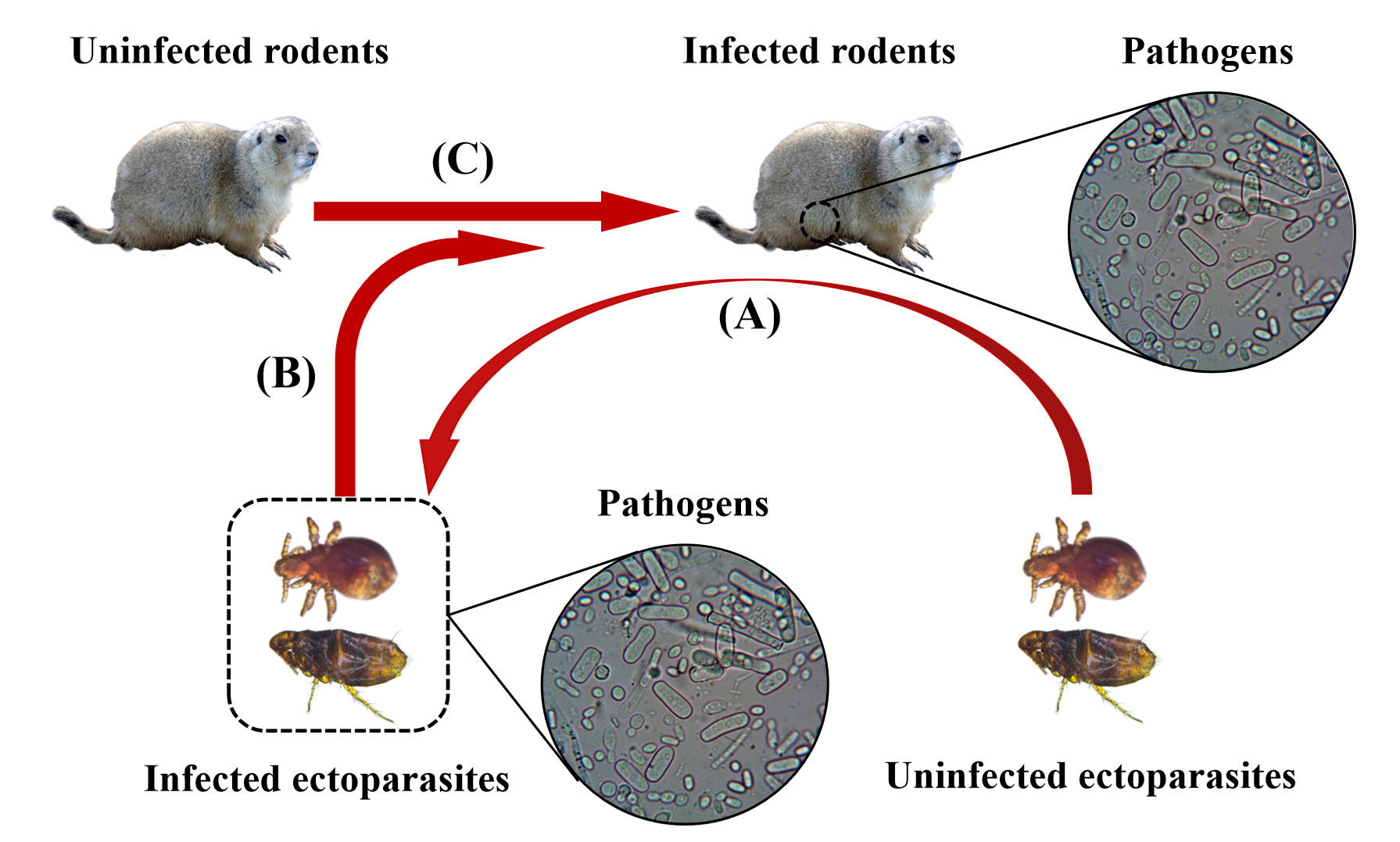

Supplement: SUPPLEMENTARY FIGURE 2 — Circulation of pathogens between ectoparasites and rodents (LTGRs): (A) Ectoparasites (e.g., fleas and lice) acquire pathogens by biting infected rodents (LTGRs); (B) Pathogen-carrying ectoparasites bite uninfected rodents (LTGRs); (C) Uninfected rodents (LTGRs) become infected by bites from pathogen-carrying ectoparasites. [file Image_2.tiff]
