# Supplementary material for: Detection of spotted fever group rickettsiae and Coxiella burnetii in long-tailed ground squirrels (Spermophilus undulatus) and their ectoparasites
Source: Front Vet Sci. 2025 Mar 6;12:1553152. doi: 10.3389/fvets.2025.1553152 (PMC11923762; doi:10.3389/fvets.2025.1553152)
Supplement: SUPPLEMENTARY TABLE 1 — Target genes and sequences of primers used in this study for identifying Long-tailed ground squirrels (LTGRs), fleas, lice, spotted fever group rickettsiae (SFGR) and Coxiella burnetii. [file Table_1.docx]

**Supplementary Table 1.** Target genes and sequences of primers used in this study for identifying Long-tailed ground squirrels (LTGRs), fleas, lice, spotted fever group rickettsiae (SFGR) and *Coxiella burnetii*

| Species/Pathogen | Gene | Primer sequence (5’-3’) | Fragment size (bp) | Reference |
| --- | --- | --- | --- | --- |
| LTGRs | *cytb* | L7: ACCAATGACATGAAAAATCATCGTT  H15915: TCTCCATTTCTGGTTTACAAGAC | 1178 bp | [19] |
| fleas | *COII* | CO11A-TLEUF: ATGGCAGATTAGTGCAATGG  CO11B-TLYSR: GTTTAAGAGACCAGTACTTG | 780 bp | [21] |
| lice | *18S rRNA* | NS1: GTAGTCATATGCTTGTCTC  NS2: CGCGGCTGCTGGCACCAGACTTGC | 570 bp | [22, 23] |
| SFGR | *ompA* out | ompA-OUTF: ATGGCGAATATTTCTCCAAAA  ompA-OUTR: AGTGCAGCATTCGCTCCCCCT | 532 bp | [17] |
|  | *ompA* in | ompA-INF: CTTAAAGCCGCTTTATTCACCACCTC  ompA-INR: CCTGTATAATTATCGGCAGGAGC | 433 bp |  |
|  | *ompB* out | ompB-OUTF: ACAGCTACCATAGTAGCCAG  ompB-OUTR: TGCAGTATAGTTACCACCG | 1063 bp | [17] |
|  | *ompB* in | ompB-INF: TGCTGCGGCTTCTACATT  ompB-INR: ACCGCCAGCGTTCCCTAT | 812 bp |  |
|  | *gltA* out | gltA-OUTF: ATGACCAATGAAAATAATAAT  gltA-OUTR: ATTGCAAAAAGTACAGTGAACA | 1178 bp | [17] |
|  | *gltA* in | gltA-INF: GGAATCTTGCGGCATCGAGGATATG  gltA-INR: CCATAGCTTTATAGATAATACCCG | 931 bp |  |
|  | *sca1* out | sca1-OUTF: GGTGATGAAGAAGAGTCTC  sca1-OUTR: CTCTTTAAAATTATGTTCTAC | 657 bp | [17] |
|  | *sca1* in | sca1-INF: GAGGTTTGTGGATGCGTGGT  sca1-INR: ACTGTGACTTTAGTACCGACA | 553 bp |  |
| *Coxiella burnetii* | *Com1* out | Com1-OUTF: AGTAGAAGCATCCCAAGCATTG  Com1-OUTR: TGCCTGCTAGCTGTAACGATTG | 501 bp | [24] |
|  | *Com1* in | Com1-INF: GAAGCGCAACAAGAAGAACA  Com1-INR: TGGAAGTTATCACGCAGTTG | 438 bp |  |
|  | *IS1111* | IS1111F: TATGTATCCACCGTAGCCAGTC  IS1111R: CCCAACAACACCTCCTTATTC | 687 bp | [25] |
