# Supplementary material for: Detection of spotted fever group rickettsiae and Coxiella burnetii in long-tailed ground squirrels (Spermophilus undulatus) and their ectoparasites
Source: Front Vet Sci. 2025 Mar 6;12:1553152. doi: 10.3389/fvets.2025.1553152 (PMC11923762; doi:10.3389/fvets.2025.1553152)
Supplement: SUPPLEMENTARY TABLE 2 — Cycling conditions of PCR assays. [file Table_2.docx]

**Supplementary Table 2.** Cycling conditions of PCR assays

| Gene | Denaturation (°C, S) | Denaturation (°C, S) | Annealing (°C, S) | Extension (°C, S) | Cycle number | Final extension (°C, S) |
| --- | --- | --- | --- | --- | --- | --- |
| *cytb* | 94, 300 | 94, 30 | 55, 60 | 72, 60 | 30 | 72, 600 |
| *COII* | 94, 240 | 94, 60 | 47, 60 | 72, 180 | 33 | 72, 300 |
| *18S rRNA* | 95, 180 | 95, 60 | 58, 60 | 72, 90 | 35 | 72, 420 |
| *ompA* out | 95, 300 | 95, 30 | 50, 30 | 72, 30 | 35 | 72, 480 |
| *ompA* in | 95, 300 | 95, 30 | 59, 30 | 72, 30 | 35 | 72, 480 |
| *ompB* out | 95, 300 | 95, 45 | 56, 45 | 72, 150 | 35 | 72, 300 |
| *ompB* in | 95, 300 | 95, 45 | 56, 45 | 72, 70 | 35 | 72, 300 |
| *gltA* out | 95, 300 | 95, 40 | 50, 40 | 72, 60 | 37 | 72, 480 |
| *gltA* in | 95, 300 | 95, 30 | 58, 30 | 72, 60 | 37 | 72, 300 |
| *sca1* out | 95, 300 | 95, 30 | 50, 30 | 72, 30 | 35 | 72, 300 |
| *sca1* in | 95, 300 | 95, 30 | 55, 30 | 72, 30 | 35 | 72, 300 |
| *Com1* out | 95, 300 | 95, 30 | 60, 30 | 72, 60 | 35 | 72, 600 |
| *Com1* in | 95, 300 | 95, 30 | 60, 30 | 72, 60 | 35 | 72, 600 |
| *IS1111* | 94, 300 | 94, 30 | 60, 30 | 72, 60 | 35 | 72, 600 |
