# Supplementary material for: Detection of spotted fever group rickettsiae and Coxiella burnetii in long-tailed ground squirrels (Spermophilus undulatus) and their ectoparasites
Source: Front Vet Sci. 2025 Mar 6;12:1553152. doi: 10.3389/fvets.2025.1553152 (PMC11923762; doi:10.3389/fvets.2025.1553152)
Supplement: SUPPLEMENTARY TABLE 3 — The BLAST analysis of Long-tailed ground squirrels (LTGRs), fleas, lice, spotted fever group rickettsiae (SFGR) and Coxiella burnetii. [file Table_3.docx]

**Supplementary Table 3.** The BLAST analysis of Long-tailed ground squirrels (LTGRs), fleas, lice, spotted fever group rickettsiae (SFGR) and *Coxiella burnetii*

| Part A. The BLAST analysis of LTGRs in this study | | | | | |
| --- | --- | --- | --- | --- | --- |
| Gene | species (Genbank accession no.) | | % sequence similarity | Host | Country |
| *cytb* | *Spermophilus undulatus* (OQ695583) | | 99.86 (1124/1137) | - | Russia |
| Part B. The BLAST analysis of fleas in this study | | | | | |
| *COII* | *Citellophilus tesquorum dzetysuensis* (PP475165) | | 100 (728/728) | *Spermophilus undulatus* | China |
|  | *Frontopsylla elatoides elatoides* (MF000677) | | 99.86 (713/714) | *Spermophilus undulatus* | China |
|  | *Neopsylla mana* (MF000670) | | 100 (727/727) | *Spermophilus undulatus* | China |
| Part C. The BLAST analysis of lice in this study | | | | | |
| *18S rRNA* | *Linognathoides urocitelli* (MK478719) | | 99.81 (522/523) | *Spermophilus undulatus* | Mongolia |
| Part D. The BLAST analysis of SFGR in this study | | | | | |
| Gene | species | SFGR (Genbank accession no.) | % sequence similarity | Host | Country |
| *ompA* | flea | *Rickettsia sibirica* (KT006594) | 100 (407/407) | *Homo sapiens* | Russia |
|  |  | *Rickettsia slovaca* (MW922580) | 100 (411/411) | *Dermacentor marginatus* | Kazakhstan |
|  |  | *Rickettsia raoultii* (MZ297809) | 99.50 (397/399) | *Apodemus agrarius* | China |
|  |  | *Rickettsia felis* (MT499365) | 100 (420/420) | *Canis lupus familiaris*, *Felis catus* | Indonesia |
|  | louse | *Rickettsia sibirica* (KT006594) | 100 (405/405) | *Homo sapiens* | Russia |
|  |  | *Rickettsia felis* (MT499365) | 99.76 (410/411) | *Canis lupus familiaris*, *Felis catus* | Indonesia |
|  | *Spermophilus undulatus* | *Rickettsia sibirica* (KT006594) | 100 (426/426) | *Homo sapiens* | Russia |
|  |  | *Rickettsia raoultii* (MZ297809) | 99.47 (375/377) | *Apodemus agrarius* | China |
|  |  | *Rickettsia felis* (MT499365) | 100 (414/414) | *Canis lupus familiaris*, *Felis catus* | Indonesia |
| *ompB* | flea | *Rickettsia sibirica* (KT006598) | 100 (906/906) | *Homo sapiens* | Russia |
|  |  | *Rickettsia slovaca* (MN388790) | 100 (987/987) | *Pipistrellus pipistrellus* | China |
|  |  | *Rickettsia raoultii* (MW922588) | 99.32 (588/592) | *Dermacentor marginatus* | Kazakhstan |
|  |  | *Rickettsia felis* (JN366420) | 99.87 (741/742) | *Ctenocephalides* spp. | Ethiopia |
|  | louse | *Rickettsia sibirica* (KT006598) | 99.55 (893/897) | *Homo sapiens* | Russia |
|  |  | *Rickettsia felis* (JN366420) | 100 (742/742) | *Ctenocephalides* spp. | Ethiopia |
|  | *Spermophilus undulatus* | *Rickettsia sibirica* (KT006598) | 100 (999/999) | *Homo sapiens* | Russia |
|  |  | *Rickettsia raoultii* (MW922588) | 99.32 (588/592) | *Dermacentor marginatus* | Kazakhstan |
|  |  | *Rickettsia felis* (JN366420) | 99.73 (740/742) | *Ctenocephalides* spp. | Ethiopia |
| *gltA* | flea | *Rickettsia sibirica* (MW802694) | 100 (891/891) | *Rhipicephalus turanicus* | China |
|  |  | *Rickettsia slovaca* (MW922558) | 100 (939/939) | *Hyalomma asiaticum* | Kazakhstan |
|  |  | *Rickettsia raoultii* (KM288477) | 99.89 (902/903) | *Dermacentor* sp. | Russia |
|  |  | *Rickettsia felis* (MT019627) | 99.89 (893/894) | *Canis lupus familiaris* | China |
|  | louse | *Rickettsia sibirica* (MW802694) | 100 (894/894) | *Rhipicephalus turanicus* | China |
|  |  | *Rickettsia felis* (MT019627) | 99.81 (1069/1071) | *Canis lupus familiaris* | China |
|  | *Spermophilus undulatus* | *Rickettsia sibirica* (MW802694) | 100 (891/891) | *Rhipicephalus turanicus* | China |
|  |  | *Rickettsia raoultii* (KM288477) | 99.89 (875/876) | *Dermacentor* sp. | Russia |
|  |  | *Rickettsia felis* (MT019627) | 99.89 (908/909) | *Canis lupus familiaris* | China |
| *sca1* | flea | *Rickettsia sibirica* (CP170616) | 99.81 (512/513) | *Homo sapiens* | China |
|  |  | *Rickettsia slovaca* (CP002428) | 100 (528/528) | - | France |
|  |  | *Rickettsia raoultii* (MG811691) | 100 (512/512) | *Dermacentor nuttalli*, *Dermacentor silvarum* | China |
|  |  | *Rickettsia felis* (AY355362) | 100 (513/513) | - | France |
|  | louse | *Rickettsia sibirica* (CP170616) | 100 (510/510) | *Homo sapiens* | China |
|  |  | *Rickettsia felis* (AY355362) | 100 (515/515) | - | France |
|  | *Spermophilus undulatus* | *Rickettsia sibirica* (CP170616) | 100 (513/513) | *Homo sapiens* | China |
|  |  | *Rickettsia raoultii* (MG811691) | 100 (519/519) | *D**ermacentor nuttalli*, *Dermacentor silvarum* | China |
|  |  | *Rickettsia felis* (AY355362) | 100 (515/515) | - | France |
| Part E. The BLAST analysis of *Coxiella burnetii* in this study | | | | | |
| Gene | species | *Coxiella burnetii* (Genbank accession no.) | % sequence similarity | Host | Country |
| *Com1* | flea | *Coxiella burnetii* (MK372916) | 100 (402/402) | *Bubalus bubalis* | India |
|  | louse | *Coxiella burnetii* (MK372916) | 100 (399/399) | *Bubalus bubalis* | India |
|  | *Spermophilus undulatus* | *Coxiella burnetii* (MK372916) | 100 (396/396) | *Bubalus bubalis* | India |
| *IS1111* | flea | *Coxiella burnetii* (MZ073364) | 99.54 (647/650) | *Rhipicephalus sanguineus* | Turkey |
|  | louse | *Coxiella burnetii* (MZ073364) | 99.69 (649/651) | *Rhipicephalus sanguineus* | Turkey |
|  | *Spermophilus undulatus* | *Coxiella burnetii* (MZ073364) | 99.69 (652/654) | *Rhipicephalus sanguineus* | Turkey |
